# Impact of Goji Berry Juice on Redox Status in Wistar Rats: A Subchronic Toxicity Assessment

**DOI:** 10.3390/ijms27020631

**Published:** 2026-01-08

**Authors:** Cristiane de Freitas Rodrigues, Jean Ramos Boldori, Félix Roman Munieweg, Marcell Valandro Soares, Bibiana Pistoia Rabuske, Thais Ribeiro Pinheiro, Cristiane Casagrande Denardin

**Affiliations:** Biochemistry and Toxicology of Bioactive Compounds Research Group, Federal University of Pampa (UNIPAMPA), Campus Uruguaiana, BR 472, Km 585, Caixa Postal 118, Uruguaiana CEP 97501-970, RS, Brazil; cristianedfr@gmail.com (C.d.F.R.); jeanboldori.aluno@unipampa.edu.br (J.R.B.); felixmunieweg@gmail.com (F.R.M.); marcellprofile@hotmail.com (M.V.S.); bibianapfr@gmail.com (B.P.R.); thaisripinheiro@gmail.com (T.R.P.)

**Keywords:** pro-oxidant, food supplement, oxidative stress

## Abstract

Goji berry consumption provides various beneficial health effects, although little is known about the possible toxicological and pro-oxidant effects. Therefore, this study aimed to evaluate the subchronic oral toxicity of goji berry juice (GBJ) for 28 days in Wistar rats (OECD 407). The GBJ was prepared in a blender with water and then filtered. The total phenolic compounds were evaluated using the Folin method (μg equivalent of gallic acid/mL juice). Forty 90-day-old female Wistar rats were divided into four groups of 10 animals each. The control group received an oral saline solution of 1 mL/100 g, and the treatments received daily doses of 1.85, 5.68, and 11.36 μg GAE/100 g for 28 days. Our findings revealed that GBJ does not alter animal body weight or food intake, although we observed higher hepatic transaminase levels and reactive species generation in the liver and kidney, which may have led to imbalanced antioxidant defenses and damaged lipids and proteins. Additionally, we observed kidney damage with increased Bowman space. Our 28-day findings indicate that goji berry juice at doses equivalent to typical human consumption can induce early redox imbalances and hepatic and renal biochemical alterations in female Wistar rats, warranting caution and further long-term, sex-inclusive studies.

## 1. Introduction

Concerns about eating and well-being have led consumers to seek alternative foods and supplements to prevent or delay health damage [1]. New products with a functional appeal or classified as dietary supplements are frequently launched on the market, albeit they are not always evaluated for their beneficial and toxicological effects [2]. Among such functional foods and supplements, the goji berry (*Lycium barbarum*), which is native to China and Asia, has spread throughout the world and is primarily sold as dehydrated fruit or as capsules [3]. 

The goji berry has attracted the interest of researchers and received functional appeals mainly due to its rich phytochemical content, such as polysaccharides, lipids, terpenes, and phenolic compounds [4]. Flavonoids represent the most abundant group of phenolic compounds in *Lycium* fruits, more notably catechin, epicatechin, apigenin, luteolin, kaempferol, quercetin, myricetin, rutin, and quercetin-rhamno-di-hexoside. Moreover, *L. barbarum* fruits have tannins, coumarins, lignans, and phenolic acids. The most abundant phenolic acids are hydroxybenzoic, gallic, syringic, vanillic, chlorogenic, caffeic, p-coumaric, and ferulic acids [4,5,6].

The main health benefits of phenolic compounds include antioxidant activity, anti-inflammatory, anti-diabetic, neuroprotective, and anti-cancer properties, and gut microbiota-modulating effects [4,5]. *Lycium* berries are also rich in other bioactive molecules, such as polysaccharides, terpenes, and carotenoids (zeaxanthin). Carbohydrates are the most abundant component (~51%) in goji berries, and the water-soluble polysaccharide fraction has been identified as an important bioactive molecule. An increasing body of evidence has shown that these polysaccharides influence blood glucose levels, body weight, and lipid metabolism, among others [7,8].

Dried goji berries are traditionally used in Chinese soups, herbal teas, tinctures, wine, juice, powders, and tablets [2,9]. Nevertheless, goji berries are more commonly consumed fresh or in beverages (e.g., juices) [2,5]. Dried *Lycium* fruits are generally incorporated into complex herb formulae in traditional medicines at doses of 6–18 g. In decoctions, doses of 5–15 g are recommended (equivalent to 25–120 g of fresh berries), while 10 g is recommended 2–3 times a day in folk medicine. For juice, 30 mL four times daily (120 mL/day), equivalent to roughly 150 g of fresh berries, has been advised [5]. 

However, only a handful of studies have investigated the adverse or toxicological effects of goji berries. Clinical reports have shown that goji berries may interact with drugs used in blood coagulation, such as warfarin [10], present hepatotoxicity [11], produce photosensitivity on the skin [12], and cause food allergies [13]. Nevertheless, a 14-day toxicological study in rats performed after daily oral administrations of goji berry juice showed no toxicity [14]. 

Numerous studies have suggested the health benefits of dietary antioxidants [15]. However, many antioxidants may also exhibit pro-oxidant behavior under certain conditions, and factors such as metal-reducing potential, chelating behavior, pH, and solubility characteristics determine if the phenolic compound activity will be antioxidant or pro-oxidant [16]. Pro-oxidants cause oxidative damage through reactive species and antioxidant system inhibition; hence, antioxidants might also have pro-oxidant behavior [17].

A recent study by our research group revealed pro-oxidant effects of goji berry juice using a *Caenorhabditis elegans* model, and we observed that goji berry juice increased reactive species, lipid oxidative damage, and parameters related to prematurely aging worms and reduced survival and longevity [18]. Given this scenario and the need for further research, this study sought to assess the pro-oxidant and toxicological effects of goji berry juice through a 28-day subchronic oral toxicity study in Wistar rats [19].

## 2. Results and Discussion

Goji berries are a source of numerous bioactive compounds and characterized by significant antioxidant and biological potential [3]. We observed that GBJ had a total phenolic compound content of 946.6 µg of gallic acid equivalents/mL (µg GAE/mL). Based on the content of total phenolic compounds, the doses used on the animals were 1.89, 5.68, and 11.36 µg GAE/g. Nonetheless, we observed several toxic effects in female Wistar rats after 28 days of consuming GBJ. Thus, this is the first study to evaluate the toxic and possible pro-oxidant effects of commercially sold goji berries, consumed as juice, mimicking the population’s daily consumption of 100 mL, 50 mL, and 20 mL of juice a day (60 kg adult). Although this study only evaluated the effect of juice consumption in females, these results should already be used to ensure that juice consumption is carried out with caution.

None of the animals died after 28 days of consuming GBJ. The animals’ weight and food consumption also remained unaltered (Table 1). Similarly, Vidal and collaborators [20] observed that young and elderly mice that consumed an aqueous lacto-wolfberry solution, which is similar to GBJ, did not experience alterations in their body weight or food intake. In addition, we did not observe any change in the relative weight of vital organs and tissues (e.g., liver, kidneys, and retroperitoneal fat); however, the spleen weight significantly increased with 1.89 µg GAE/g compared to the control (Table 1).

The spleen maintains the number of blood cells and has an immunological and iron recycling function [21,22]. An increase in the function of this organ can increase its size, which is known as splenomegaly. This change can occur in response to stress or a chronic process harmful to the individual [22], in addition to being caused by various diseases and drugs that affect the liver and cause hematological toxicity [23,24,25]. Evidence has shown that aniline exposure results in iron overload and induced oxidative stress in the spleen, which may cause transcriptional upregulation of fibrogenic/inflammatory cytokines via activation of oxidative stress-responsive signaling pathways [26]. Thus, it is plausible that the splenomegaly observed in GBJ-treated animals may be caused by increased oxidative stress in this organ or possible hematological damage.

As for the blood biochemical analysis, 28 days of GBJ consumption did not alter the serum parameters of glucose, cholesterol, triglycerides, albumin, creatinine, bilirubin, and ALP compared to the control (Table 1). Nevertheless, the AST levels significantly increased in animals treated with 1.89 and 11.36 µg GAE/g, as did the ALT levels in animals treated with 1.89 and 5.68 µg GAE/g compared to control (Table 1). Clinical diagnosis of diseases and damage to the liver’s structural integrity is generally assessed by monitoring the status of serum AST, ALP, and ALT activities, which are sensitive serological indicators of liver toxicity [27].

**Figure 1:**
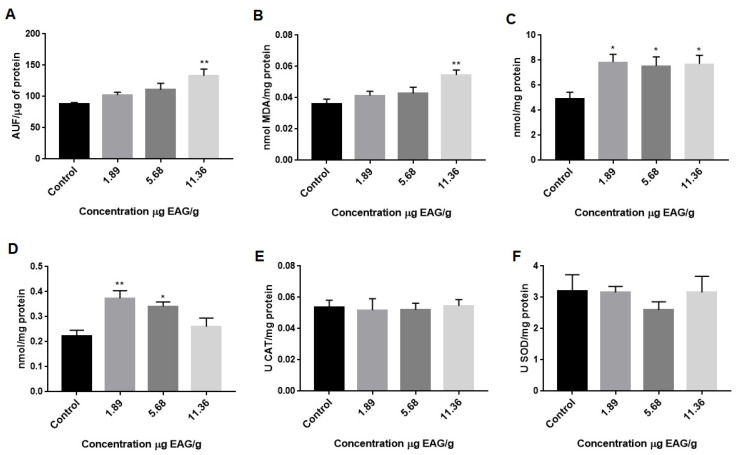
Goji berry (*Lycium barbarum* L.) juice increases oxidative stress and oxidative damage in rat livers. (**A**) Reactive species (DCF); (**B**) lipidic peroxidation (TBARS); (**C**) protein carbonylation; (**D**) non-protein thiols (SHNP); (**E**) superoxide dismutase (SOD) activity; (**F**) catalase activity. Values are expressed as mean ± standard deviation of at least ten independent animals. * Difference in control * (*p* < 0.05) ** (*p* < 0.01).

El-Bakry et al. [28] investigated the effects of consuming green tea extract for 30 days in rats and found significantly higher ALT and AST levels due to this plant’s hepatotoxicity. Hence, our findings suggest that consuming GBJ may damage the liver of animals, which may be due to increased oxidative stress in this organ. Given that we found changes in liver blood markers caused by GBJ consumption, we evaluated the oxidative damage parameters and activity of this organ’s primary antioxidant enzymes (Figure 1). As illustrated in Figure 1A, the consumption of 11.36 µg GAE/g significantly increases reactive species production in the liver. Reactive oxygen species generation plays a critical role in liver damage and initiating hepatic fibrogenesis [29]. When the amount of pro-oxidant substances exceeds the cellular antioxidant capacity, oxidative stress occurs, leading to potential cell damage [29]. Such oxidative damage generated by GBJ consumption was found in the liver by increased lipid peroxidation at 11.36 µg GAE/g and increased protein carbonylation at all GBJ concentrations (Figure 1B,C). Thus, it is highly plausible that GBJ has pro-oxidant effects at the concentrations tested in this study.

One finding that reinforces this pro-oxidant effect of GBJ is the significant increase in glutathione (GSH) levels, which was estimated by the non-protein thiol content in the liver, which was significantly higher at the lowest GBJ concentrations (1.89 and 5.68 µg GAE/g) and reduced at levels of control of 11.36 µg GAE/g (Figure 1D). The GSH, a principal antioxidant in the cell, is a tripeptide present in high amounts in all cells, including hepatocytes. It is synthesized in the cytosol in a two-step energy-consuming process and distributed to different organelles, including the endoplasmic reticulum and mitochondria [29]. Therefore, an increase in GSH levels did not occur at concentrations with greater pro-oxidant effects. We also observed that catalase and superoxide dismutase activities in the liver were not affected by GBJ consumption (Figure 1E,F).

Arroyo-Martinez et al. [12] reported a case of hepatotoxicity caused by goji berry consumption; a 60-year-old woman showed symptoms of abdominal discomfort and blood analysis revealed elevated ALT and AST levels. According to a friend’s recommendation, the woman had been consuming goji berry tea three times a day for ten days (a handful of berries for each cup) but no other medication. This is one of the few reports of toxicity by goji berries, and no study has evaluated the pro-oxidant effects of this fruit. Similar to our findings, there are reports of toxicity from other Chinese fruits, such as *Averrhoa carambola* L., which is also known as star fruit and Chinese gooseberry [30]. The researchers found that rats exposed to *A. carambola* juice extract for 28 days were apparently unharmed, although subacute administration of the extract led to kidney and liver injuries, especially at the highest dose (600 mg/kg body weight). The authors also observed higher ALT and AST levels in the blood and increased lipid peroxidation assessed by MDA levels [30].

**Figure 2:**
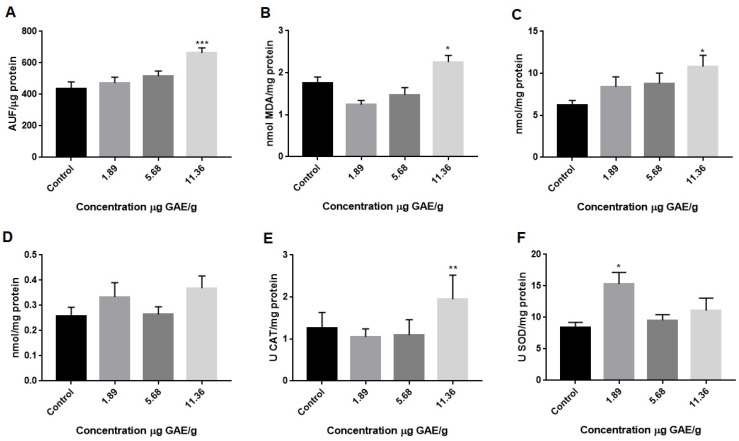
Goji berry (*Lycium barbarum* L.) juice increases oxidative stress and oxidative damage in rat kidneys. (**A**) Reactive species (DCF); (**B**) lipidic peroxidation (TBARS); (**C**) protein carbonylation; (**D**) non-protein thiols (SHNP); (**E**) superoxide dismutase (SOD) activity; (**F**) catalase activity. Values are expressed as mean ± standard deviation of at least ten independent animals. * Difference in control * (*p* < 0.05) ** (*p* < 0.01) *** (*p* < 0.001).

The kidney is a vital organ that controls homeostasis, water volume, electrolytes in the blood, and blood pressure, its main function being urine filtration. Oxidative stress has a part in the pathophysiology of renal impairment and is a mediator of chronic kidney disease progression. Furthermore, during substitutive therapy with hemodialysis or peritoneal dialysis and in case of transplantation, the organism continues to be exposed to oxidation, causing the development of major systemic comorbidities, particularly cardiovascular diseases [31]. Our results also demonstrated that consuming 11.36 µg GAE/g of GBJ for 28 days significantly increased reactive species, lipid peroxidation, and protein carbonylation in the kidney (Figure 2A–C). Additionally, the levels of non-protein thiols remained unchanged (Figure 2D). As for catalase and superoxide dismutase activity, GBJ increased their activity at 11.36 and 1.89 µg GAE/g, respectively (Figure 2E,F). 

Once again, our findings show a possible pro-oxidant effect of GBJ in rodents. Data have demonstrated that substances considered antioxidants may have harmful effects under certain conditions [32,33]. The pro-oxidant activity of carotenoids depends on their interaction with biological membranes and co-antioxidants (e.g., vitamin C) [34]. Even flavonoids have been reported to act as pro-oxidants in systems containing transition metals. Flavonoids, such as quercetin and kaempferol, induce DNA damage and lipid peroxidation in the presence of the transition metal. Moreover, phenolics can also display pro-oxidant effects, especially in a system containing redox-active metals [33]. 

Our research group recently identified various phenolic compounds in GBJ [18]. From the hydroxybenzoate derivatives, we observed the presence of catechin as a major compound, followed by vanillic and protocatechuic acids and small amounts of gallic acid. P-coumaric and trans-ferulic acids were observed in hydroxycinnamate derivatives, and the only major flavonoid found was rutin [18]. The pro-oxidating effects of GBJ were also demonstrated when we used *C. elegans* as an experimental model. In this study, GJB increased reactive species and lipid peroxidation and promoted the premature aging of worms [18]. As observed in *C. elegans*, the phenolic compounds in GBJ may also participate in reactions that produce reactive oxygen species and other organic radicals that damage DNA, lipids, and other biological molecules in mammals (e.g., rats and humans).

**Figure 3:**
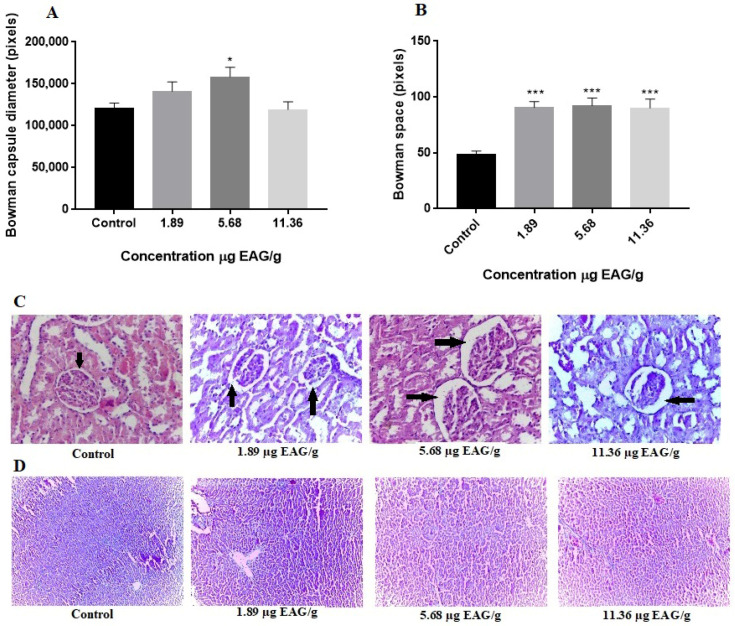
Histological analyses of the liver and kidneys of rats treated with goji berry (*Lycium barbarum* L.) juice. (**A**) Bowman capsule size; (**B**) Bowman space. Representative photos of the kidney (**C**) and liver. Arrows indicate Bowman’s space. (**D**). Values are expressed as mean ± standard deviation of at least ten independent animals. * Difference in control * (*p* < 0.05) *** (*p* < 0.001).

The kidneys excrete harmful substances from the body, and even the slightest alterations may cause toxicant accumulation and tissue damage [35]. The Bowman capsule is a component of the renal corpuscle, the origin of the urinary tubules that constitute the kidney’s nephrons. The glomerulus is a collection of capillaries lined by a delicate mesangial matrix, while the Bowman’s capsule consists of two epithelium layers: visceral (envelops glomerulus) and parietal. Bowman’s capsule is responsible, together with the glomerulus, for the first kidney blood filtration phase [36,37]. Our findings also showed that GBJ promoted histological changes in the rats’ kidneys, including increasing the diameter of Bowman’s capsule and Bowman’s space (Figure 3); this was likely due to glomerular hypertrophy. Factors such as aging and diet are common elements that influence the effectiveness of glomerular filtration, and this scenario is related to various human diseases such as diabetes, oligomeganephronia, and obesity, among others [36]. Therefore, excreting GBJ may lead to glomerular hyperfiltration, which may promote these dysfunctions in animals. Histological evaluations of the rats’ livers revealed no apparent changes, although we observed intense reactive species production and damage to lipids and proteins in this organ (Figure 3). We did not observe any histological changes in the liver, although we did observe changes in oxidative status. We believe that as the liver has great potential for regeneration, the oxidative damage observed was not capable of altering the architecture and macroscopic characteristics of the organ.

## 3. Materials and Methods

### 3.1. Goji Berry Juice

Two batches of dried goji berries (Grade Jasmine^®^, São Paulo, Brazil) were purchased locally in Uruguaiana (RS, Brazil). The goji berry juice (GBJ) was prepared by adding 100 g of fruit to 900 mL of distilled water and blending in a mixer for 10 min, and the juice was then filtered through a sieve, aliquoted, and frozen (−20 °C) until analyses [20].

The total phenolic compound content of the juice was quantified using the Folin–Ciocalteu method [38]. Briefly, the procedure consisted of transferring 100 µL standard or sample into a 2 mL polypropylene tube, followed by additions of 1600 µL milli q H_2_O and 100 µL Folin–Ciocalteu reagent. After mixing the samples and incubating for 3 min, 200 µL 1N Na_2_CO_3_ was added. The sample mixtures were allowed to stand for 1 h at room temperature. Aliquots of 200 µL were transferred to clear microtitre wells in duplicate and the absorbance was measured at 725 nm using a M5 microplate reader (Molecular Devices, San Jose, CA, USA). A gallic acid standard curve was used for quantification.

The doses used for the toxicological test were based on the recommended consumption of juice for humans (100 mL per day), which is equivalent to 1 mL/100 g for rodents per day [5]. The doses used were 1, 0.5, and 0.2 mL juice/100 g of animal/day. Based on calculations of equivalent doses for humans, these doses would be the equivalent of a 60 kg adult consuming 100 mL, 50 mL, and 20 mL of juice a day. To express the results, these values were standardized by expressing these volumes in the total phenolic compound content: 1.89, 5.68, and 11.36 µg GAE/g. 

### 3.2. Experimental Animals and Subchronic Toxicity Study

Forty female Wistar rats weighing 200–250 g were used and maintained in the bioterium of the Federal University of Pampa (Uruguaiana, Brazil). The animals were fed commercial feed (Presence^®^, São Paulo, Brazil) and kept in plastic cages (40 × 32 × 16 cm) at room temperature (22 ± 1 °C) with relative humidity (60 ± 10%) and a 12:12 day/night cycle. The experiment was approved by the animal ethics committee of the Federal University of Pampa (CEUA no. 043/2018).

The oral subchronic toxicity study was conducted according to Organization for Economic Co-operation and Development (OECD) Guideline 407 [19]. The animals were randomly divided into four groups of 10 animals each. Each animal of the control group received 1 mL saline/100 g per day, and the other three groups received oral gavage doses of 1, 0.5, and 0.2 mL GBJ/100 g for 28 consecutive days. The animals’ weight and food consumption were assessed weekly.

### 3.3. Serum Biochemical Analysis

On the twenty-ninth day of the experiment and before the subchronic oral toxicity tests, the animals were fasted for 8 h and anesthetized by intraperitoneal injection of ketamine 10% (100 mg/kg) and xylazine 3% (50 mg/kg). Blood (6–9 mL) was collected by cardiac puncture in tubes with separator gel, and serum was used to analyze the total cholesterol, triglycerides, glucose, albumin, urea, creatinine, uric acid, total bilirubin, conjugate bilirubin, alkaline phosphatase (ALP), alanine transaminase (ALT), and aspartate aminotransferase (AST). This analysis utilized standard laboratory kits (Bioclin, Belo Horizonte, Brazil) according to the manufacturer’s instructions.

### 3.4. Tissue Preparation and Oxidative Stress Analyses

The kidneys, liver, spleen, and retroperitoneal fat were removed and weighed. The organs’ relative weight was expressed as the percentage of body weight. The tissues were washed in cold 0.9% saline and kept in ice until used to prepare homogenates. Liver and kidney samples were homogenized in 150 mM saline and centrifuged at 10,000× *g* for 10 min at 4 °C; the homogenate and supernatant were used to assess oxidative stress. 

Reactive species analyses were conducted using the dichlorodihydrofluorescein diacetate assay, which consists of analyzing the conversion of dichloro-dihydro-fluorescein diacetate (DCFH-DA) to 2–7‘dichlorofluorescein [39]. In summary, the reaction was carried out using 10 µL of homogenate (liver or kidney) plus 180 µL of 150 mM saline, 10 µL DCFH-DA, and 10 µL of 0.4 mM H_2_O_2_. After 30 min of incubation at room temperature, the fluorescence was measured using a microplate reader (M5, Molecular Devices, San Jose, CA, USA) at 535 nm excitation and 485 nm emission. The results were expressed as AUF/mg of protein.

Non-protein thiol (NPSH) levels in the liver and kidney were determined according to Ellman [40] and expressed as µmol/mg of protein. The lipid peroxidation in the liver and kidney was assessed using the thiobarbituric acid reactive substance (TBARS) assay, as described by Ohkawa et al. [41]. The results were expressed as nmol/mg protein.

Protein carbonylation content in the liver and kidney was measured using a slightly modified version of the method of Levine et al. [42]. A sample (0.2 mL) was added to 0.8 mL of 10 mM 2,4-dinitrophenylhydrazine or 2 M HCl, and the samples were incubated in the dark for 1 h and homogenized every 15 min. The samples were precipitated with 1 mL of 20% trichloroacetic acid (TCA) and centrifuged at 10,000 rpm at 4 °C for 10 min; the same procedure was repeated once with 10% TCA. The precipitate was washed thrice with 1 mL ethanol/ethyl acetate (1:1). The precipitate was then resuspended in 2% SDS at 37 °C. Protein carbonylation content was calculated from the maximum absorbance (370 nm) using a molar extinction coefficient of 22,000 M^−1^ cm^−1^. The results were expressed as nmol carbonyl per mg protein. The samples’ protein content was determined using the method of Lowry [43] and bovine serum albumin as the standard.

### 3.5. Antioxidant Enzyme Measurements

Copper, zinc-superoxide dismutase (Cu,Zn-SOD) activity was measured according to Misra and Fridovich [44], which consists of Cu, Zn-SOD inhibiting the auto-oxidation of adrenalin to adrenochrome at alkaline pH. Enzymatic activity was expressed as U SOD/mg protein at 32 °C (one unit, U = sample quantity that inhibits adrenaline oxidation to adrenochrome by 50%). Catalase (CAT) activity was determined using the technique of Aebi [45] and hydrogen peroxide as the substrate; H_2_O_2_ consumption was monitored spectrophotometrically at 240 nm. Enzymatic activity was expressed as U CAT/mg protein at 37 °C.

### 3.6. Histopathological Analysis

The liver and kidneys were quickly dissected, and tissue sections (5 mm) were fixed by immersion at room temperature in 10% formalin solution (*v*/*v*). Paraffin-embedded tissue sections were stained with hematoxylin–eosin (H&E) for histological examinations. The tissue samples were examined and photographed under a light microscope to observe structural abnormality. Glomerulus diameter and Bowman’s space width measurements were performed using ImageJ software version 1.54 (Wayne Rasband, National Institutes of Health, Bethesda, MD, USA).

### 3.7. Statistical Analysis

Each group comprised ten rats. One-way analysis of variance was performed, and Tukey’s post hoc test was used for multiple comparisons. Statistical analyses were performed using GraphPad 7.0 software. All experiments were performed in triplicate, and the results are expressed as mean ± standard deviation.

## 4. Conclusions

Given the above, our findings suggest that GBJ has pro-oxidant effects when consumed in doses usually used by the population (1/2 glass 100 mL/day), promoting an increase in oxidative stress and liver and kidney oxidative damage. Blood hepatic markers also presented alterations, albeit we did not see any visible histological changes in the liver. We found increased oxidative stress, oxidative damage to lipids and proteins, and relevant histological changes in the animals’ kidneys. Hence, we recommend precaution when consuming GBJ since there are reports of hepatotoxicity in the literature.

Surprisingly, many foods known as excellent antioxidants may have undesirable pro-oxidant effects, and fruits and plants are not always beneficial and harmless to health just because they are natural. Our findings revealed that GBJ increased AST and ALT levels and reactive species generation in the liver and kidney, which may have imbalanced antioxidant defenses and damaged lipids and proteins, in addition to the kidney damage and increased Bowman space reported herein. It should be emphasized that the data obtained are the results of a toxicological test of only 28 days and only with female rats. Further long-term studies with both sexes should be carried out to prove the findings of this study. Thus, this study serves as a warning for the unrestrained consumption of goji berries, as they may induce the pro-oxidant and toxic effects observed when consumed at a usual dose by rodents. 

## Figures and Tables

**Table 1:** Body parameters, food consumption, and blood biochemical analyses of animals.

	Control	1.89 µg EAG/g	5.68 µg EAG/g	11.36 µg EAG/g
Initial weight (g)	222.72 ± 9.50	211.90 ± 14.47	212.50 ± 13.90	211.00 ± 13.59
Final weight (g)	256.90 ± 11.84	251.70 ± 13.44	250.20 ± 20.34	250.00 ± 16.26
Weight gain (g)	34.17 ± 11.33	39.89 ± 10.30	37.70 ± 7.83	39.00 ± 7.23
Food consumption (g/day)	18.64 ± 1.94	18.99 ± 0.96	20.30 ± 1.69 *	19.20 ± 1.27
Liver weight (g)	7.84 ± 0.93	7.48 ± 0.51	7.30 ± 0.93	7.36 ± 0.70
Kidney weight (g)	1.74 ± 0.09	1.71 ± 0.18	1.70 ± 0.25	1.69 ± 0.12
Spleen weight (g)	0.72 ± 0.06	0.82 ± 0.10 *	0.70 ± 0.09	0.72 ± 0.06
Retroperitoneal fat weight (g)	2.69 ± 0.76	2.17 ± 0.58	2.51 ± 0.53	2.73 ± 0.58
Glucose levels (mg/dL)	103.50 ± 34.83	104.20 ± 49.25	91.30 ± 42.21	99.50 ± 27.41
Cholesterol levels (mg/dL)	110.80 ± 15.14	108.50 ± 19.58	113.50 ± 19.76	99.94 ± 15.75
Triglycerides levels (mg/dL)	220.10 ± 37.13	185.40 ± 40.96	177.40 ± 35.26	177.30 ± 58.15
Albumin levels (g/dL)	8.98 ± 2.52	9.34 ± 2.98	9.21 ± 2.38	9.02 ± 2.89
Creatinine levels (mg/dL)	0.59 ± 0.14	0.41 ± 0.31	0.48 ± 0.27	0.53 ± 0.15
Bilirubin levels (mg/dL)	4.30 ± 1.41	3.92 ± 1.16	4.72 ± 1.38	4.58 ± 1.83
Alkaline phosphatase levels (U/L)	105.00 ± 39.51	73.74 ± 28.88	88.74 ± 30.44	100.90 ± 35.94
Aspartate aminotransferase (AST) levels (U/L)	152.20 ± 21.81	287.10 ± 126.00 **	197.10 ± 82.24	252.00 ± 85.15 **
Alanine Aminotransferase (ALT) levels (U/L)	43.32 ± 7.87	69.38 ± 22.95 **	135.00 ± 51.83 ***	78.97 ± 50.22

Results expressed as mean ± SD. * indicate difference from control * (*p* < 0.05); ** (*p* < 0.01); *** (*p* < 0.001).

## Data Availability

The datasets used and analyzed during the current study are available on request.
